# Effects of High-Order Interactions among *IGFBP-3* Genetic Polymorphisms, Body Mass Index and Soy Isoflavone Intake on Breast Cancer Susceptibility

**DOI:** 10.1371/journal.pone.0162970

**Published:** 2016-09-15

**Authors:** Qiong Wang, Li Liu, Hui Li, Ping Tao, Yana Qi, Jiayuan Li

**Affiliations:** 1 Department of Health Policy and Management, School of Public Health, Sun Yat-sen University, Guangzhou, Guangdong, China; 2 Department of Epidemiology and Biostatistics, West China School of Public Health, Sichuan University, Chengdu, Sichuan, China; 3 Comprehensive Guidance Center of Women's Health, Chengdu Women’s and Children’s Central Hospital, Chengdu, Sichuan, China; 4 Department of Breast Surgery, the Second People’s Hospital of Sichuan Province, Chengdu, Sichuan, China; 5 West China Hospital, Sichuan University, Chengdu, Sichuan, China; University of North Carolina at Chapel Hill School of Medicine, UNITED STATES

## Abstract

**Background:**

Polymorphisms of *IGF-1* and *IGFBP-3* and environmental factors may work together to influence insulin-like growth factor (IGF) levels and thus breast cancer (BC) risk. However, very few studies have investigated high-order interactions among these variables.

**Methods:**

A total of 277 newly diagnosed BC cases and 277 controls were recruited between October 2010 and July 2012. We collected each participant's demographic characteristics, dietary intake, and blood sample. *IGF-1 rs1520220* and *IGFBP-3 rs2854744* were then genotyped. A multi-analytic strategy combining unconditional logistic regression (ULR), generalized multifactor dimensionality reduction (GMDR), and classification and regression tree (CART) approaches was applied to systematically identify the interactions of the two single nucleotide polymorphisms (SNPs), body mass index (BMI), and daily intake of soy isoflavone (DISI) on BC susceptibility.

**Results:**

In GMDR analyses, high-order interactions among BMI, DISI, and SNP *rs2854744* were identified among overall and postmenopausal women. We also found significant dosage effects on BC risk with an increasing number of exposure factors, namely carrying the *rs2854744* AA genotype, DISI <9.85 mg/day, and BMI ≥24 kg/m^2^ (*P*_trend_<0.05). Similarly, in CART analyses, compared with individuals having BMI<24kg/m^2^, DISI<9.85 mg/day, and the *rs2854744* CC+CA genotype, BC risk increased significantly for those carrying the *rs2854744* AA genotype, with BMI<24 kg/m^2^ and DISI<9.85 mg/day (OR = 1.95, 95%CI: 1.03–3.69), and also for those with BMI≥24kg/m^2^ and DISI<9.85 mg/day (OR = 2.13, 95%CI: 1.00–4.51). Similar interaction effects were observed among postmenopausal women.

**Conclusions:**

This study suggests high-order interactions of the *IGFBP-3 rs2854744 AA* genotype, BMI≥24kg/m^2^, and DISI<9.85 mg/day on increased BC risk, particularly among postmenopausal women.

## Introduction

Breast cancer (BC) is common among women worldwide, with about1.67 million new cases diagnosed in 2012, accounting for 25% of all cancers [1]. In China, the incidence of BC has steadily risen, from 126,227 in 2002 [2] to over 169,000 in 2008 [3], to 187,213 in 2012 [1]. Unless this trend slows, BC cases in China are expected to reach 2.5 million overall by 2021[4].

The insulin-like growth factor (IGF) system plays important roles in cellular proliferation, differentiation, and apoptosis [5–7]; therefore, the IGF system has long been known to be involved in BC carcinogenesis. The IGF system mainly consists of IGF-1/IGF-1 receptors, IGF-2/IGF-2 receptors, and IGF binding proteins (IGFBP-1 to 6). Within the IGF system, IGF-1 and IGFBP-3 are two key subunits involved in carcinogenesis.

Although the relationship between circulating IGFBP-3 concentration and BC remains inconsistent [8–11], high circulating IGF-1 levels have been observed in many in vivo and epidemiological studies to increase the risk and progression of BC [12–15]. Thus, determinants of circulating IGF-1 and IGFBP-3 levels, including genetic and environmental factors, may impact BC risk.

Among genetic factors, the G-C substitution in intron 3 of the *IGF-1* gene (SNP *rs1520220*) may influence IGF-1 expression by altering the secondary structure of RNA or DNA [16, 17], and the A-C substitution at nucleotide-202 in the promoter region of the *IGFBP-3* gene (SNP *rs2854744*) may reduce promoter activity, which would theoretically decrease circulating IGFBP-3 levels [18].

Apart from genetic polymorphisms, other factors may also play roles in serum IGF-1 or IGFBP-3 variation. Obesity is a known risk factor for BC [19]. Body mass index (BMI) has shown effects on circulating IGF-1 or IGFBP-3 concentrations. Fowke et al. observed IGFBP-3 levels tend to rise with BMI, regardless of race[20]. As for dietary factors, soy isoflavone is a type of phytoestrogen with similar molecular structure to endogenous estrogens. It is believed that high soy isoflavone intake contributes to the relatively low BC risk in Asian countries [21]. Evidence from in vitro studies indicates that genistein, a main component of soy isoflavones, may stimulate the IGF-1 signaling pathway in human breast cancer cells at pharmacological doses [22]. Given that genetic and environmental factors are associated with circulating IGF levels, the hypothesis that *IGF-1* and *IGFBP-3* polymorphisms, BMI, and dietary intake of soy isoflavone may work together in affecting BC risk warrants further investigation. Hakkak et al. found obese rats fed soy exhibited a significant decrease in IGFBP-3 levels [23]. Our previous study observed joint effects of carrying *IGF-1rs1520220* and consuming soy isoflavone on women's circulating IGF-1 levels [24]. However, to the best of our knowledge, no previous study has examined potential interactions of *IGF-1* and *IGFBP-3* polymorphisms, BMI, and dietary soy isoflavone on BC risk. To test for these interactions, we conducted a case control study to (i) identify the gene-environment interactions of IGF-1 *rs1520220*, IGFBP-3 *rs2854744*, BMI, and soy isoflavone intake on BC risk; and (ii) estimate the effects of gene-environment interactions.

## Materials and Methods

### Ethics Statement

This study was approved by the institutional research ethics committee of Sichuan University, and written informed consent was obtained from each subject before completing the questionnaire survey and laboratory tests.

### Population

From October 2010 to July 2012, 292 primary BC cases newly histopathologically diagnosed in the Second People’s Hospital of Sichuan Province (also known as Sichuan Cancer Hospital) were invited to participate in our study, among whom, 15 cases (5%) were excluded because their blood samples were not available. A total of 277 cases were enrolled, of which 7 were diagnosed with breast carcinoma in situ (DCIS), 246 with invasive ductal carcinoma, and 24 with other cancers (e.g., invasive lobular carcinoma, medullary carcinoma). According to pathologic reports and using the American Joint Committee on Cancer (AJCC) TNM System (0, I, II, III, IV, and unknown stage or not applicable), 20 cases were classified as stage 0, 87 as stage I, 91 as stage II, 40 as stage III, 2 as stage IV, and 37 as unknown. During the same period, 306 women undergoing routine physical examinations in Chengdu Women’s and Children’s Central Hospital were selected as potential controls. They were then given breast ultrasound to exclude malignant tumors; however, those with benign breast disease, such as lobular hyperplasia, were included. Each control was matched to one patient by age (±2 years), so that 277 healthy women (90.5% of the potential control group) were included in our study as controls. All participants were of Han ethnicity and had lived in Sichuan Province for more than 20 years. We further excluded those with occupational exposure, other malignant tumors, or psychiatric disorders.

### Data collection

We used a structured questionnaire to collect all participants’ socio-demographic and reproductive characteristics, and a semi-quantitative dietary questionnaire to collect their long-term (≥5 years) dietary habits. Evaluation of the reliability and structural validity of the questionnaires and calculation of energy-adjusted dietary intake has been described in detail in our previous study [25]. In brief, we calculated the total daily intake of energy first, then used a residual method to adjust other dietary intake as energy-adjusted protein, fat, carbohydrate, dietary fiber, and daily intake of soy isoflavones (DISI). According to the Chinese Dietary Reference Intakes (DRIs) (formulated by the Chinese DRIs committee in 2000) for 18–50 year old women with moderate physical activity [26], we used the following dichotimization: 2300 kcal/day for total energy, 70 g/day for protein, and 77 g/day for fat. For those categories without recommended levels of dietary intake, the mean of dietary intakes (132.52 g/day for carbohydrate, 17.86 g/day for dietary fiber, and 9.85 mg/day for soy isoflavones) were selected as the cutoff values of high vs. low intake.

### Genotype analyses

Five milliliters of whole blood was obtained from each participant via venipuncture into an anticoagulative tube and stored at -20°Cuntil DNA extraction. Genomic DNA was extracted from whole blood using a TIANamp Blood DNA Kit (TIANGEN, Beijing). DNA samples with purity between 1.8 and 2.0 qualified for genotyping. *IGF-1 rs152022*0 and *IGFBP-3 rs2854744* were genotyped with TaqMan assays, which were performed with an ABI 7500 thermal cycler (Applied Biosystems, Foster City, CA). Primers and probes for *IGF-1 rs1520220* were purchased as predesigned assays-on-demand from Applied Biosystems (ABI assay-on-demand C_2801118_10). For *IGFBP-3 rs2854744*, primers (forward: CACCTTGGTTCTTGTAGACGACAA; reverse: GGCGTGCAGCTCGAGACT) and probes (VIC-MGB-TCCTCGTGCGCACG and FAM-MGB-CTCGTGCTCACGCC) were used. All tests were performed in the molecular biology laboratory of West China School of Public Health, Chengdu, China. In addition, 5% of the total subjects were selected randomly for duplicate testing, and the determined genotypes of repeated tests were in complete concordance.

### Statistical analyses

For each SNP, we checked the Hardy-Weinberg equilibrium (HWE) among all controls via a goodness-of-fit chi-square test. Differences in demographic characteristics, reproductive and dietary factors between cases and controls were compared with independent-sample T-tests (for continuous variables) or chi-square/Fisher's exact tests (for categorical variables). We applied multivariable unconditional logistic regression (ULR) to test the main effects of SNPs *IGF-1 rs1520220* and *IGFBP-3 rs2854744*, and the joint effects of SNPs and BMI, and of SNPs and DISI on BC risk, by calculating odds ratios (ORs) and 95% confidence intervals (95% CIs). The Akaike Information Criterion (AIC) was used to determine the goodness of model fit.

According to the results of previous studies, IGF-1 levels were significantly lower for *IGF-1 rs1520220* CC genotype carriers than for GC or GG carriers [27], and carrying the *IGFBP-3 rs2854744* AA genotype was associated with higher circulating IGFBP-3 levels [28]. We therefore analyzed the effects of *IGF-1 rs1520220* with CC vs. GC+GG and *IGFBP-3 rs2854744* with AA vs. CC+CA. SPSS18.0 was used for statistical analyses.

### GMDR analyses

We applied generalized multifactor dimensionality reduction (GMDR, version 0.9, obtained from http://www.ssg.uab.edu/gmdr/) to analyze possible high-order interactions among genetic and environmental factors, obtaining parameters such as balanced accuracy, sign test *P* value, and cross-validation (CV) consistency. The model with the maximum balanced accuracy, the maximum CV consistency score, and a *P* value of 0.05 or less was considered the best. The odds ratios (OR) with 95% confidence intervals (95% CIs) for the interaction effects of the variables in the best model were calculated using ULR analysis by classifying subjects into groups according to the number of exposure risk factors, and adjusting potential confounders as covariates [29].

### CART analyses

CART analysis was performed with SPSS18.0. Via recursive partitioning, a CART is constructed by splitting a node into two child nodes step by step, beginning with the root node that contains the whole learning sample and ending with a decision tree. Before building a tree, a Gini criterion was used to choose the measurement for goodness of split that would yield the maximum homogeneity between two child nodes. Tree splitting was done until terminal nodes reached a pre-specified minimum size of 30 subjects. To avoid overfitting, a pruning procedure would be performed when a tree had grown to its full depth. Terminal nodes of the tree represent the subgroups with differential risk associations with BC, indicating the potential presence of interactions. Finally, the risk for these subgroups was evaluated using the ULR by treating the subgroup having the smallest percentage of cases as the reference and adjusting for potential confounder factors as covariates [30].

All the data analyses were also performed stratified by menopausal status.

## Results

### General demographic characteristics, related reproductive factors and dietary intake of study subjects

Among the participants, the 227 cases included 143 pre- and 134 postmenopausal women, while there were 187 pre- and 90 postmenopausal controls. Even though the menopausal status was not exactly equal between the two groups, the difference of mean (SD) ages between cases (49.29±11.04) and controls (47.77±9.04) was not significant (*t* = -1.76, *P* = 0.08).

Among all study participants (Table 1) as well in the pre- and postmenopausal subgroups, distributions of education, income, age at first pregnancy, parity, and breast feeding were significantly different between cases and controls (*P*<0.05). Cases tended to be less educated and have lower income, younger age at first pregnancy, more children and longer breast feeding than controls. BMI was also significantly different between cases and controls in the overall and postmenopausal groups. These factors were treated as priori chosen potential confounders and adjusted in ULR, GMDR and CART analyses. The ratios of women who ever used contraceptives and had a family history of BC were higher among postmenopausal women than premenopausal women.

**Table 1:** Demographic characteristics, reproductive factors and breast cancer among study participants.

Factors	Cases (%)	Controls (%)	*P*
**Education**			
**Less than high school**	192 (69.3)	62 (22.4)	**<0.001**
**High school or more**	85 (30.7)	215 (77.6)	
**Income (RMB per month)**			
**<1500**	203 (73.3)	96 (34.7)	**<0.001**
**≥1500**	74 (26.7)	181 (65.3)	
**BMI (kg/m**^**2**^**)**			
**<24**	187 (67.5)	222 (80.1)	**0.001**
**≥24**	90 (32.5)	55 (19.9)	
**Smoking**			
**No**	272 (98.2)	271 (97.8)	0.76
**Yes**	5 (1.8)	6 (2.2)	
**Alcohol consumption**			
**No**	265 (95.7)	265 (95.7)	1.00
**Yes**	12 (4.3)	12 (4.3)	
**Menarche (years)**			
**≥13**	232 (83.8)	225 (81.2)	0.43
**<13**	45 (16.2)	52 (18.8)	
**Age at first pregnancy (years)**			
**<25**	189 (68.2)	121 (43.7)	**<0.001**
**≥25**	88 (31.8)	156 (56.3)	
**Parity**			
**0**	3 (1.1)	6 (2.2)	
**1–2**	224 (80.9)	265 (95.7)	**<0.001**
**≥3**	50 (18.1)	6 (2.2)	
**No. of abortions**			
**0**	57 (20.6)	57 (20.6)	
**1–2**	148 (53.4)	164 (59.2)	0.24
**≥3**	72 (26.0)	56 (20.2)	
**Breast feeding (months)**			
**≥3**	237 (85.6)	190 (68.6)	**<0.001**
**<3**	40 (14.4)	87 (31.4)	
**Contraceptive use**			
**Never**	222 (80.1)	227 (81.9)	0.59
**Ever**	55 (19.9)	50 (18.1)	
**History of benign breast disease**			
**No**	229 (82.7)	217 (78.3)	0.20
**Yes**	48 (17.3)	60 (21.7)	
**Family history of breast cancer**			
**No**	266 (96.0)	269 (97.1)	0.48
**Yes**	11 (4.0)	8 (2.9)	

Table 2 shows the dietary intake of our subjects. Among study participants, energy-adjusted protein, fat, dietary fiber, and DISI were significantly different between cases and controls (*P*<0.05). However, the results stratified by menopausal status had some differences. There were significant differences in energy-adjusted protein, fat, carbohydrate, and dietary fiber intake between premenopausal cases and controls, while for postmenopausal subjects, the only significant differences were in DISI consumption.

**Table 2:** Dietary intake and breast cancer by menopausal status.

Dietary intake	Total			Premenopausal			Postmenopausal		
	Cases (%)	Controls (%)	χ2 *(P)*	Cases (%)	Controls (%)	χ2 *(P)*	Cases (%)	Controls (%)	*χ2 (P)*
**Total energy intake (kcal/day)**									
**<2300**	246 (88.8)	241 (87.0)		124 (86.7)	160 (85.6)		122 (91.0)	81 (90.0)	
**≥2300**	31 (11.2)	36 (13.0)	0.42 (0.52)	19 (13.3)	27 (14.4)	0.09 (0.77)	12 (9.0)	9 (10.0)	0.07 (0.79)
**Protein intake (g/day)**^**a**^									
**<70**	239 (86.3)	203 (73.3)		125 (87.4)	134 (71.7)		114 (85.1)	69 (76.7)	
**≥70**	38 (13.7)	74 (26.7)	**14.50 (<0.001)**	18 (12.6)	53 (28.3)	**11.91 (0.001)**	20 (14.9)	21 (23.3)	2.55 (0.11)
**Fat intake(g/day)** ^**a**^									
**<77**	51 (18.4)	99 (35.7)		23 (16.1)	76 (40.6)		28 (20.9)	23 (25.6)	
**≥77**	226 (81.6)	178 (64.3)	**21.06 (<0.001)**	120 (83.9)	111(59.4)	**23.27 (<0.001)**	106 (79.1)	67 (74.4)	0.67 (0.42)
**Carbohydrate intake(g/day)** ^**a**^											
**<132.52**	152(54.9)	137(49.5)		83(58.0)	80(42.8)		69(51.5)	57(63.3)	
**≥132.52**	125(45.1)	140(50.5)	1.63(0.20)	60(42.0)	107(57.2)	**7.55(0.006)**	65(48.5)	33(36.7)	3.07(0.08)
**Dietary fiber intake(g/day)** ^**a**^									
**<17.86**	165(59.6)	133(48.0)		86(60.1)	80(42.8)		79(59.0)	53(58.9)	
**≥17.86**	112(40.4)	144(52.0)	**7.44(0.01)**	57(39.9)	107(57.2)	**9.77(0.002)**	55(41.0)	37(41.1)	0.001(0.99)
**DISI (mg/day)** ^**a**^									
**≥9.85**	112(40.4)	135(48.7)		59(41.3)	84(44.9)		53(39.6)	51(56.7)	
**<9.85**	165(59.6)	142(51.3)	**3.87 (0.049)**	84(58.7)	103(55.1)	0.44(0.51)	81(60.4)	39(43.3)	**6.34(0.01)**

^a:^ The dietary key nutrient intake values, including protein, fat, carbohydrates, dietary fiber, and soy isoflavones, were adjusted for energy by the residual method

### *IGF-1 rs1520220* and *IGFBP-3 rs28 54744* genotypes and BC

Among all controls, the frequencies of the C allele for *IGF-1 rs1520220* and the C allele for *IGFBP-3 rs2854744* were 55.8% and 25.0%, respectively. Genotypes of *IGF-1 rs1520220* and *IGFBP-3 rs2854744* accorded with HWE (*IGF-1 rs1520220*: *χ*^2^ = 3.63, *P* = 0.06; *IGFBP-3 rs2854744*: *χ*^2^ = 0.56, *P* = 0.45). The main effects of *IGF-1 rs1520220* and *IGFBP-3 rs2854744* on BC are shown in the S1 Table. Results from multivariable ULR analysis showed that after adjustment for confounders, *IGF-1 rs1520220* and *IGFBP-3 rs2854744* were not associated with BC risk.

### Joint effects of *IGF-1 rs1520220, IGFBP-3 rs2854744* and BMI or DISI

Joint effects of *IGF-1 rs1520220*, *IGFBP-3 rs2854744* and BMI are shown in the S2 Table. Significant joint effects were mainly found among postmenopausal women. We observed that when using the *IGF-1* GG+GC genotype and BMI<24 kg/m^2^ as the reference group, carrying the *IGF-1* GG+GC genotype with BMI ≥24 kg/m^2^ increased BC risk. Compared with women with the *IGFBP-3* CC+CA genotype and BMI<24 kg/m^2^, other groups (*IGFBP-3* AA& BMI<24 kg/m^2^; *IGFBP-3* CC+CA& BMI ≥24 kg/m^2^; *IGFBP-3* AA& BMI≥24 kg/m^2^) had a significantly higher risk for BC. Also, we found carrying the *IGFBP-3* AA genotype worked jointly with low soy intake (DISI <9.85 mg/day) to increase BC risk among postmenopausal women (S3 Table).

### GMDR analyses

To further explore gene-environment interactions, we performed a GMDR analysis (S4 Table). A three-factor interaction model of BMI, DISI, and *IGFBP-3 rs2854744* was identified as the best model among overall and postmenopausal women, with the maximum balanced accuracy for the training set (58.48% for overall and 66.56% for postmenopausal women), balanced accuracy for the calibration set (58.09% for overall and 64.51% for postmenopausal women), the maximum CV consistency of 10/10, and a sign test *P* value of 0.01 and 0.001 for overall and postmenopausal women, respectively. The results indicated potential interactions among BMI, DISI, and *IGFBP-3 rs2854744*. The interaction effects were then estimated using ULR analyses (Table 3). Subjects were classified into four subgroups by the number of exposure factors, defined as the *IGFBP-3 rs2854744* AA genotype, DISI<9.85 mg/day, and BMI ≥24 kg/m^2^. A significant dose-response relationship was observed for BC risk among overall and postmenopausal women (*P*_trend_ is 0.015 for overall and <0.001 for postmenopausal women). Compared with subjects carrying no exposure factors, those with two risk factors had higher BC risk (for overall women: OR = 1.73, 95%CI: 0.98–3.06; for postmenopausal women: OR = 4.96, 95%CI: 1.94–12.66), and the risk increased further for those carrying three factors (for overall women: OR = 2.74, 95%CI: 1.25–6.03; for postmenopausal women: OR = 5.76, 95%CI: 1.62–20.45).

**Table 3:** Cumulative effects of *IGFBP-3 rs2854744*, DISI, and BMI on breast cancer risk.

NO. ^a^	Total			Premenopausal			Postmenopausal		
	Cases (%)	Controls (%)	OR (95%CI)^b^	Cases (%)	Controls (%)	OR (95%CI)^c^	Cases (%)	Controls (%)	OR (95%CI)^d^
**0**	28(10.1)	36(13.0)	1.00	18(12.6)	20(10.7)	1.00	10(7.5)	16(17.8)	1.00
**1**	101(36.5)	140(50.5)	0.93(0.53–1.62)	57(39.9)	91(48.7)	0.70(0.34–1.43)	44(32.8)	49(54.4)	1.44(0.59–3.49)
**2**	116(41.9)	86(31.0)	**1.73(0.98–3.06)**	54(37.8)	66(35.3)	0.91(0.44–1.89)	62(46.3)	20(22.2)	**4.96(1.94–12.66)**
**3**	32(11.6)	15(5.4)	**2.74(1.25–6.03)**	14(9.8)	10(5.3)	1.56(0.56–4.36)	18(13.4)	5(5.6)	**5.76(1.62–20.45)**
***P***_**trend**_	**0.015**			0.489			**<0.001**		

^a^: The number of exposure factors, defined as theIGFBP-3 rs2854744 AA genotype, DISI <9.85 mg/day, and BMI ≥24 kg/m2

^b^: adjusted for education, income, age at first pregnancy, parity, breast feeding, and energy-adjusted protein, fat and dietary fiber intake

^c:^ adjusted for education, income, age at first pregnancy, parity, breast feeding, and energy-adjusted protein, fat, carbohydrate and dietary fiber intake

^d^: adjusted for education, income, age at first pregnancy, parity, breast feeding, and contraceptive use

### CART analyses

**Fig 1:**
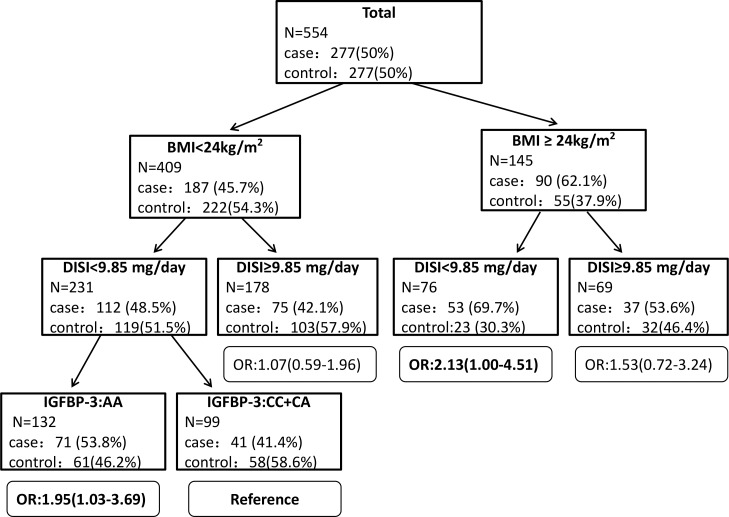
CART analysis of *IGF-1* and *IGFBP-3* genetic polymorphisms and environmental factors among study participants. After CART analysis, the risk for subgroups identified in different terminal nodes was evaluated using the ULR by treating the subgroup having the smallest percentage of cases as the reference and adjusting for potential confounder factors, including education, income, age at first pregnancy, parity, breast feeding, and energy-adjusted protein, fat, and dietary fiber intake. The results of ULR, ORs, and their 95% confidence intervals for each subgroup are shown by the side of each terminal node of the tree.

To further validate the gene-environment interactions defined by GMDR, we performed classification and regression tree (CART) analyses. **Fig 1** depicts the resulting tree structure generated for study participants. There was an initial split on BMI, confirming that BMI was the most important risk factor for BC among the factors considered. With the smallest percentage of cases (41.4%), the subgroup with BMI<24kg/m^2^, DISI<9.85 mg/day and the *IGFBP-3 rs2854744* CC+CA genotype was treated as a reference. Compared with the reference subgroup, BC risk was significantly higher for those with the *IGFBP-3 rs2854744* AA genotype, BMI<24 kg/m^2^, and DISI<9.85 mg/day (OR = 1.95, 95%CI: 1.03–3.69), and for those with BMI≥24 kg/m^2^, DISI<9.85 mg/day and either *IGFBP-3 rs2854744* genotype (OR = 2.13, 95%CI: 1.00–4.51). However, we did not observe significant gene-environment interactions among the variables considered in premenopausal women (data not shown).

**Fig 2:**
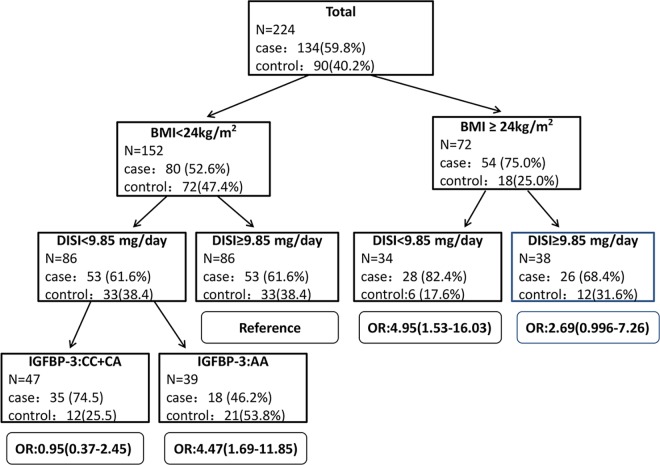
CART analysis of *IGF-1* and *IGFBP-3* genetic polymorphisms and environmental factors among postmenopausal women. This figure summarizes the results of CART analysis for postmenopausal women. The risk for subgroups was also evaluated using ULR with adjustment for education, income, age at first pregnancy, parity, breast feeding, and estrogen use. The results of ULR, ORs, and their 95% confidence intervals for each subgroup are shown by the side of each terminal node of the tree.

The results for postmenopausal women are summarized in **Fig 2**. Here the tree also split initially on BMI, and the reference group was that with BMI<24kg/m^2^ and DISI≥9.85 and either *IGFBP-3 rs2854744* genotype. Compared with the reference, the BC risk for those with BMI≥24kg/m^2^ was higher (OR = 2.69, 95%CI: 0.996–7.26), and the risk was still higher in the subgroups with both BMI≥24 kg/m^2^ and DISI<9.85 mg/day (OR = 4.95, 95%CI: 1.53–16.03), and both DISI<9.85 mg/day and the *IGFBP-3 rs2854744* AA genotype (OR = 4.47, 95%CI: 1.69–11.85).

## Discussion

In this study, we applied a multiple-pronged strategy combining ULR, GMDR, and CART analyses to systematically examine the association between BC risk and a series of risk factors. These included SNPs of *IGF-1 rs1520220* and *IGFBP-3 rs2854744*, BMI, and soy isoflavone intake. Results from the GMDR and CART analyses consistently revealed a high-order interaction of the *IGFBP-3 rs2854744* genotype, BMI, and DISI on BC risk. Having the *IGFBP-3 rs2854744 AA* genotype, BMI≥24 kg/m^2^, and DISI<9.85 mg/day may synergistically increase women's BC risk, particularly among postmenopausal women.

### *IGF-1 rs1520220, IGFBP-3 rs2854744,* and BC

Given the association between high levels of circulating IGF-1 and increased risk and progression of BC, it is believed that genetic polymorphisms associated with serum IGF-1 variation can also affect BC risk. Numerous epidemiologic studies have examined the relationship between genes encoding IGF-1and BC risk (reviewed in [28]), but with respect to SNP *IGF-1 rs1520220*, the results are inconsistent. For example, Al-Zahrani et al. reported that women carrying the C allele of *IGF-1 rs1520220* had a 1.41-fold higher BC risk [31], while Qian et al. observed that this SNP predicted circulating IGF levels but not breast cancer risk among Chinese women [27]. As in Qian et al.’s study, we did not detect a significant relationship between SNP *IGF-1 rs1520220* and BC risk. The differing results can be explained by heredity. A SNP database at the National Center of Biotechnology Information reveals that the *IGF-1 rs1520220* C allele frequency is 45.8–63.3% among Asians (55.8% in our study), much less than that among Europeans (75.0–100%) [32].

Although the A allele of *IGFBP-3 rs2854744* is positively associated with circulating IGFBP-3 levels, with a distinct dose-response relationship [33–35], there remains conflicting evidence about the association between *IGFBP-3 rs2854744* and BC risk. A study in Europe with 8,760 subjects reported that women carrying the A allele of *IGFBP-3* had an 87% lower risk [31]. A case-control study from Shanghai of 2,503 women showed a 1.6-fold higher risk conferred by the *IGFBP-3* C allele [36]. However, several studies have found no association between this SNP and breast cancer risk [27, 28, 33, 37], including the present study. In vitro studies found that *IGFBP-3* gene expression varied by approximately 50% between A- and C- containing alleles, whereas circulating levels varied according to genotype to a lesser extent (7.7%) [33]. Since this SNP has only weak effects on circulating IGFBP-3 level, it is not easy to detect a significant relationship between the *IGFBP-3 rs2854744* genotype alone and BC risk.

### High-order interactions among *IGFBP-3* genetic polymorphisms, body mass index, and soy isoflavone intake on BC risk

Although we did not observe effects of *IGF-1 rs1520220* and *IGFBP-3 rs2854744* alone on BC, we did find joint effects of *IGF-1 rs1520220* and BMI, *IGFBP-3 rs2854744* and BMI, and *IGFBP-3 rs2854744* and DISI using multivariable ULR. To further explore possible high-order gene-environment interactions, we performed GMDR and CART analyses and consistently obtained the most interesting findings in this study, suggesting there were high-order gene-environment interactions of BMI, DISI, and *IGFBP-3 rs2854744* on BC risk among overall and postmenopausal women. Furthermore, ULR analyses indicated that BC risk was associated with three risk factors in a dose dependent manner: the *IGFBP-3 rs2854744* AA genotype, DISI <9.85 mg/day, and BMI ≥24 kg/m^2^. The ORs for interaction effects among these factors ranged from 1.73 to 2.74 for overall and from 2.69 to 5.76 for postmenopausal women. These results are biologically plausible since the *IGFBP-3 rs2854744* AA genotype, DISI<9.85 mg/day, and BMI ≥24 kg/m^2^ may work together to increase circulating IGFBP-3 levels, which has been observed to be positively associated with the risk of BC among Chinese women [10]. Some researchers have observed that IGFBP-3 levels tend to rise with BMI [20, 38, 39]. Deal et al. demonstrated a synergetic effect of BMI>27 kg/m^2^ and carrying the *IGFBP-3 rs2854744*-A allele on increasing IGFBP-3 levels [33].The protective effect of dietary soy intake against breast cancer has been demonstrated by a number of studies in Asia (reviewed in [40]). Hakkaket al. found obese rats fed with soy exhibited a significant decrease in IGFBP-3 levels [23]. Population studies suggested there was trend toward decreased IGFBP-3 concentrations in women with increasing isoflavone consumption [41, 42]. We therefore suggest that increased IGFBP-3 level maybe a key mediator of the association between BC risk, the *IGFBP-3 rs2854744* AA genotype, DISI<9.85 mg/day, and BMI ≥24 kg/m^2^ in our study population.

It is notable that the interactions were limited to postmenopausal women. Postmenopausal women are more susceptible to the effects of soy isoflavone [43], an exogenous phytoestrogen, because of their sharply decreased hormone levels. Moreover, postmenopausal women have a higher average BMI than that of premenopausal women [44]. Therefore, interactions of soy isoflavone intake, BMI, and gene polymorphisms may be more easily detected among postmenopausal women. However, the exact underlying mechanisms for the differences in interaction effects between pre- and postmenopausal women remain to be elucidated.

### Strengths

There are two main strengths of our study. First, to the best of our current knowledge, this is the first study exploring the complex gene-environment interactions of *IGF-1 rs1520220* and *IGFBP-3 rs2854744* polymorphisms, BMI, and soy isoflavone intake on BC susceptibility. Moreover, we used complex statistical analyses, including GMDR and CART, to explore high-order gene-environment interactions. Compared with logistic regression, GMDR and CART are high powered for identifying high-order interactions [45, 46].These methods have been applied widely to explore high-order gene-gene and gene-environment interactions on cancer risk [29, 47, 48]. However, GMDR and CART are non-parametric data mining approaches, with a disadvantage in estimating ORs and 95% CIs for interaction effects. We thus used ULR to calculate interaction effects of variables defined in the best model of GMDR and those defined by CART. We obtained similar estimations of interaction effects based on GMDR (Table 1) and CART (**Fig 1** and **Fig 2)**. Thus, we believe our results are robust.

### Limitations

This study has several limitations. First, the cases and controls were not closely matched in general demographic characteristics, related reproductive factors and dietary intake (Table 1 and Table 2). In particular, the results showing that cases had a younger age at first pregnancy, more children, and longer breast feeding time than controls were contrary to established knowledge [49].This may be because cases were from both urban (78%) and rural areas (22%) while controls were only from urban areas, which may have introduced selection bias. However, we adjusted for potential confounders in our analyses to reduce the bias, making it highly likely that the high-order gene-environment interaction results in our study are valid.

Second, our subjects were exclusively Chinese women, so the results may not be generalizable to women from other countries with different ethnicities, dietary habits, and lifestyles. However, our study results add a new clue on the effects of gene-environment interactions on BC susceptibility.

Third, the sample size in this study was limited, which may lead to unstable results. Since the estimates of interaction based on different methods were similar, we believe our results are robust, and not likely to be influenced by the relatively small sample size.

## Conclusions

In conclusion, our study explored complex gene-environment interactions among genetic polymorphisms of the IGF system, BMI, and soy isoflavone intake on BC susceptibility. The results showed that having the *IGFBP-3 rs2854744 AA* genotype, BMI≥24 kg/m^2^, and DISI<9.85 mg/day may synergistically increase women’s BC risk, particularly among postmenopausal women. Our results have public health implications, suggesting that losing weight and increasing soy isoflavone intake may reduce BC risk for women with a susceptible *IGFBP-3 rs2854744* genotype.

## Supporting Information

S1 Table Associations of *IGF-1 rs1520220* and *IGFBP-3 rs2854744* with breast cancer. (DOCX)

S2 Table Joint effects of *IGF-1 rs1520220*, *IGFBP-3 rs2854744*, and BMI on breast cancer risk. (DOCX)

S3 Table Joint effects of *IGF-1 rs1520220*, *IGFBP-3 rs2854744*, and DISI on breast cancer risk. (DOCX)

S4 Table GMDR models of high-order interactions on breast cancer risk. (DOCX)
