# Supplementary material for: Effects of High-Order Interactions among IGFBP-3 Genetic Polymorphisms, Body Mass Index and Soy Isoflavone Intake on Breast Cancer Susceptibility
Source: PLoS One. 2016 Sep 15;11(9):e0162970. doi: 10.1371/journal.pone.0162970 (PMC5024997; doi:10.1371/journal.pone.0162970)
Supplement: S1 Table — (DOCX) [file pone.0162970.s001.docx]

**S1Table. Associations of *IGF-1 rs1520220* and *IGFBP-3 rs2854744* with breast cancer**

| Genotypes | Total | | |  | Premenopausal | | |  | Postmenopausal | | |
| --- | --- | --- | --- | --- | --- | --- | --- | --- | --- | --- | --- |
|  | Cases (%) | Controls (%) | OR(95%*CI*)^a^ |  | Cases (%) | Controls (%) | OR(95%*CI*)^b^ |  | Cases (%) | Controls (%) | OR(95%*CI*)^c^ |
| *IGF-1(rs1520220)* |  |  |  |  |  |  |  |  |  |  |  |
| GG+GC | 190 (68.6) | 183 (66.1) | 1.00 |  | 99 (69.2) | 124 (66.3) | 1.00 |  | 91 (67.9) | 59 (65.6) | 1.00 |
| CC | 87 (31.4) | 94 (33.9) | 0.70 (0.45-1.09) |  | 44 (30.8) | 63 (33.7) | 0.57 (0.31-1.05) |  | 43 (34.4) | 31 (34.4) | 0.67 (0.37-1.19) |
| *IGFBP-3(rs2854744)* |  |  |  |  |  |  |  |  |  |  |  |
| CC+CA | 103 (37.2) | 117 (42.2) | 1.00 |  | 56 (39.2) | 74 (39.6) | 1.00 |  | 47 (35.1) | 43 (47.8) | 1.00 |
| AA | 174 (62.8) | 160 (57.8) | 1.26 (0.83-1.91) |  | 87 (60.8) | 113 (60.4) | 0.82 (0.47-1.45) |  | 87 (64.9) | 47 (52.2) | 0.76 (0.44-1.30) |
| ^a^: adjusted for education, income, BMI, age at first pregnancy, parity, breast feeding, energy-adjusted protein, fat, and dietary fiber intake; ^b^: adjusted for education, income, age at first pregnancy, parity, breast feeding, energy-adjusted protein, fat, carbohydrate, and dietary fiber intake; ^c^: adjusted for education, income, BMI, age at first pregnancy, parity, breast feeding, contraceptive use, and family history of breast cancer | | | | | | | | | | | |
