# Supplementary material for: Effects of High-Order Interactions among IGFBP-3 Genetic Polymorphisms, Body Mass Index and Soy Isoflavone Intake on Breast Cancer Susceptibility
Source: PLoS One. 2016 Sep 15;11(9):e0162970. doi: 10.1371/journal.pone.0162970 (PMC5024997; doi:10.1371/journal.pone.0162970)
Supplement: S2 Table — (DOCX) [file pone.0162970.s002.docx]

**S2 Table. Joint effects of *IGF-1 rs1520220*, *IGFBP-3 rs2854744*and BMI on breast cancer risk**

| Genotypes | BMI  (kg/m^2^) | Total | | | |  | Premenopausal | | | |  | Postmenopausal | | | |
| --- | --- | --- | --- | --- | --- | --- | --- | --- | --- | --- | --- | --- | --- | --- | --- |
|  |  | Cases (%) | Controls (%) | OR(95%*CI*)^a^ | *P* _trend_ |  | Cases (%) | Controls (%) | OR(95%*CI*)^b^ | *P* _trend_ |  | Cases (%) | Controls (%) | OR(95%*CI*)^c^ | *P* _trend_ |
| *IGF-1(rs1520220)* |  | | | |  |  |  |  |  |  |  |  |  |  |  |
| GG+GC | <24 | 126 (45.5) | 147(53.1) | 1.00 | 0.53 |  | 74 (51.7) | 100 (53.5) | 1.00 | 0.25 |  | 52 (38.8) | 47 (52.2) | 1.00 | 0.07 |
| CC | <24 | 61 (22.0) | 75 (27.1) | 0.71 (0.43-1.17) |  |  | 33 (23.1) | 50 (26.7) | 0.54 (0.27-1.07) |  |  | 28 (20.9) | 25 (27.8) | 1.31 (0.54-3.16) |  |
| GG+GC | ≥24 | 64 (23.1) | 36 (13.0) | 1.44 (0.81-2.53) |  |  | 25 (17.5) | 24 (12.8) | 0.84 (0.37-1.89) |  |  | 39 (29.1) | 12 (13.3) | **3.15 (1.22-8.15)** |  |
| CC | ≥24 | 26 (9.4) | 19 (6.9) | 0.96 (0.44-2.07) |  |  | 11 (7.7) | 13 (7.0) | 0.56 (0.18-1.77) |  |  | 15 (11.2) | 6 (6.7) | 1.47 (0.46-4.74) |  |
| *IGFBP-3(rs2854744)* | |  |  |  |  |  |  |  |  |  |  |  |  |  |  |
| CC+CA | <24 | 69 (24.9) | 94 (33.9) | 1.00 | **0.059** |  | 41 (28.7) | 57 (30.5) | 1.00 | 0.60 |  | 28 (20.9) | 37 (41.1) | 1.00 | **0.008** |
| AA | <24 | 118 (42.6) | 128(46.2) | 1.29 (0.79-2.05) |  |  | 66 (46.2) | 93 (49.7) | 0.73 (0.39-1.39) |  |  | 52 (38.8) | 35 (38.9) | **2.66 (1.15-6.14)** |  |
| CC+CA | ≥24 | 34 (12.3) | 23 (8.3) | 1.37 (0.66-2.88) |  |  | 15 (10.5) | 17 (9.1) | 0.64 (0.22-1.82) |  |  | 19 (14.2) | 6 (6.7) | **4.26 (1.20-15.16)** |  |
| AA | ≥24 | 56 (20.2) | 32 (11.6) | 1.82 (0.96-3.44) |  |  | 21 (14.7) | 20 (10.7) | 0.86 (0.34-2.15) |  |  | 35 (26.1) | 12 (13.3) | **3.77 (1.36-10.45)** |  |

^a^: adjusted for education, income, age at first pregnancy, parity, breast feeding, energy-adjusted protein, fat, and dietary fiber intake; ^b^: adjusted for education, income, age at first pregnancy, parity, breast feeding, energy-adjusted protein, fat, carbohydrate, and dietary fiber intake; ^c^: adjusted for education, income, BMI, age at first pregnancy, parity, breast feeding, contraceptive use, and family history of breast cancer
