# Supplementary material for: Effects of High-Order Interactions among IGFBP-3 Genetic Polymorphisms, Body Mass Index and Soy Isoflavone Intake on Breast Cancer Susceptibility
Source: PLoS One. 2016 Sep 15;11(9):e0162970. doi: 10.1371/journal.pone.0162970 (PMC5024997; doi:10.1371/journal.pone.0162970)
Supplement: S3 Table — (DOCX) [file pone.0162970.s003.docx]

**S3 Table. Joint effects of *IGF-1 rs1520220*, *IGFBP-3 rs2854744,* and DISI on breast cancer risk**

| Genotypes | DISI  (mg/day) | Total | | | |  | Premenopausal | | | |  | Postmenopausal | | | |
| --- | --- | --- | --- | --- | --- | --- | --- | --- | --- | --- | --- | --- | --- | --- | --- |
|  |  | Cases (%) | Controls (%) | OR(95%*CI*)^a^ | *P* _trend_ |  | Cases (%) | Controls (%) | OR(95%*CI*)^b^ | *P* _trend_ |  | Cases (%) | Controls (%) | OR(95%*CI*)^c^ | *P* _trend_ |
| *IGF-1(rs1520220)* |  |  |  |  |  |  |  |  |  |  |  |  |  |  |  |
| GG+GC | ≥9.85 | 75 (27.1) | 91 (32.9) | 1.00 | 0.61 |  | 39 (27.3) | 59 (31.6) | 1.00 | 0.87 |  | 36 (26.9) | 32 (35.6) | 1.00 | 0.12 |
| CC | ≥9.85 | 37 (13.4) | 44 (15.9) | 0.76 (0.40-1.44) |  |  | 20 (14.0) | 25 (13.4) | 0.60 (0.24-1.51) |  |  | 17 (12.7) | 19 (21.1) | 0.84 (0.30-2.31) |  |
| GG+GC | <9.85 | 115 (41.5) | 92 (33.2) | 1.41 (0.86-2.32) |  |  | 60 (42.0) | 65 (34.8) | 0.95 (0.48-1.87) |  |  | 55 (41.0) | 27 (30.0) | 1.62 (0.71-3.70) |  |
| CC | <9.85 | 50 (18.1) | 50 (18.1) | 0.92 (0.50-1.71) |  |  | 24 (16.8) | 38 (20.3) | 0.52 (0.22-1.20) |  |  | 26 (19.4) | 12 (13.3) | 1.92 (0.67-5.48) |  |
| *IGFBP-3(rs2854744)* | |  |  |  |  |  |  |  |  |  |  |  |  |  |  |
| CC+CA | ≥9.85 | 41 (14.8) | 51 (18.4) | 1.00 | 0.07 |  | 22 (15.4) | 30 (16.0) | 1.00 | 0.58 |  | 19 (14.2) | 21 (23.3) | 1.00 | **0.01** |
| AA | ≥9.85 | 71 (25.6) | 84 (30.3) | 1.02 (0.55-1.89) |  |  | 37 (25.9) | 54 (28.9) | 0.58 (0.25-1.36) |  |  | 34 (25.4) | 30 (33.3) | 1.67 (0.60-4.65) |  |
| CC+CA | <9.85 | 62 (22.4) | 66 (23.8) | 1.05 (0.55-2.00) |  |  | 34 (23.8) | 44 (23.5) | 0.61 (0.25-1.48) |  |  | 28 (20.9) | 22 (24.4) | 1.46 (0.51-4.22) |  |
| AA | <9.85 | 103 (37.2) | 76 (27.4) | 1.68 (0.91-3.11) |  |  | 50 (35.0) | 59 (31.6) | 0.68 (0.30-1.56) |  |  | 53 (39.6) | 17 (18.9) | **4.10 (1.40-12.01)** |  |
| ^a^: adjusted for education, income, age at first pregnancy, parity, breast feeding, energy-adjusted protein, fat, and dietary fiber intake; ^b^: adjusted for education, income, age at first pregnancy, parity, breast feeding, energy-adjusted protein, fat, carbohydrate, and dietary fiber intake; ^c^: adjusted for education, income, BMI, age at first pregnancy, parity, breast feeding, contraceptive use, and family history of breast cancer | | | | | | | | | | | | | | | |
