# Supplementary material for: Effects of High-Order Interactions among IGFBP-3 Genetic Polymorphisms, Body Mass Index and Soy Isoflavone Intake on Breast Cancer Susceptibility
Source: PLoS One. 2016 Sep 15;11(9):e0162970. doi: 10.1371/journal.pone.0162970 (PMC5024997; doi:10.1371/journal.pone.0162970)
Supplement: S4 Table — (DOCX) [file pone.0162970.s004.docx]

**S4 Table. GMDR models of high order interactions on breast cancer risk**

| **Models** | **Balanced accuracy for training set** | **Balanced accuracy for calibration set** | ***P*** | **Cross-validation consistency** |
| --- | --- | --- | --- | --- |
| **Total ^a^** |  |  |  |  |
| BMI | 0.5632 | 0.5650 | 0.05 | 10/10 |
| BMI, IGF-1 | 0.5634 | 0.5482 | 0.17 | 9/10 |
| BMI, DISI, IGFBP-3 | **0.5848** | **0.5809** | **0.01** | **10/10** |
| **Premenopausal ^b^** |  |  |  |  |
| BMI | 0.5303 | 0.4718 | 0.38 | 7/10 |
| BMI, DISI | 0.5473 | 0.4670 | 0.95 | 7/10 |
| IGF-1, IGFBP-3, DISI | 0.5702 | 0.4713 | 0.99 | 5/10 |
| **Postmenopausal ^c^** |  |  |  |  |
| BMI | 0.6042 | 0.5149 | 0.17 | 7/10 |
| BMI, DISI | 0.6168 | 0.5756 | 0.01 | 8/10 |
| BMI, DISI, IGFBP-3 | **0.6656** | **0.6451** | **0.001** | **10/10** |
| ^a^: adjusted for education, income, age at first pregnancy, parity, breast feeding, energy-adjusted protein, fat, and dietary fiber intake; ^b^: adjusted for education, income, age at first pregnancy, parity, breast feeding, energy-adjusted protein, fat, carbohydrate, and dietary fiber intake; ^c^: adjusted for education, income, age at first pregnancy, parity, breast feeding, and contraceptive use | | | | |
